# A Case of Papillary Urothelial Neoplasm of Low Malignant Potential (PUNLMP) in Childhood

**DOI:** 10.1155/2020/8865741

**Published:** 2020-11-14

**Authors:** Reza Abbasion, Amir Jafarpisheh, Paria Dehghanian, Mahdi Mottaghi

**Affiliations:** ^1^Department of Pediatric Urology, Faculty of Medicine, Akbar Hospital, Mashhad University of Medical Sciences, Mashhad, Iran; ^2^Department of Urology, Faculty of Medicine, Mashhad University of Medical Sciences, Mashhad, Iran; ^3^Pathology Department, Pediatric Pathologist, Akbar Children's Hospital, Iran; ^4^Students Research Committee, Faculty of Medicine, Mashhad University of Medical Sciences, Mashhad, Iran

## Abstract

Urothelial carcinoma (UC) of the bladder is exceedingly rare in the pediatric population. It commonly presents as isolated hematuria. Considering the age group, the physician's low index of suspicion causes a delay in diagnosis. We present a seven-year-old girl complaining of dysuria and painless, intermittent hematuria. She was misdiagnosed with urinary tract infection several times. Although the initial ultrasound showed no abnormality, the second ultrasound after one year detected the tumor. The confirmation and resection are simultaneously achieved by cystoscopy. We concluded that chemotherapy is unnecessary due to the tumor's low-grade nature and the absence of detrusor involvement. One-year follow-up showed no relapse.

## 1. Introduction

Urothelial carcinoma (UC) of the bladder is exceedingly rare in the pediatric population. The disease incidence is about 0.1-0.4% for patients younger than twenty years old [1]. It is the most common bladder tumor between 13 and 18 years [1, 2]. Based on a recent systematic review, only 243 patients were reported in the literature until February 2019. The male to female ratio is 2 : 1 for the pediatric population [1]. We report another case of pediatric UC, which was managed successfully with tumor resection and no chemotherapy.

## 2. Case Presentation

A seven-year-old Caucasian girl referred to our tertiary clinic complaining of dysuria and painless gross hematuria of a three-month duration. She experienced three dysuria episodes last year, but further evaluation showed normal findings on ultrasound and only microscopic hematuria on urine analysis. She was treated for urinary tract infection several times, with partial improvement of her symptoms. In the last episode, the dysuria accompanied gross hematuria for a three-month duration. Repeated ultrasound showed “polyploid, 20∗29 mm mass with vascular peduncle on the right side of the bladder trigone. Size and the cortical echo of both kidneys were normal.”

**Figure 1:**
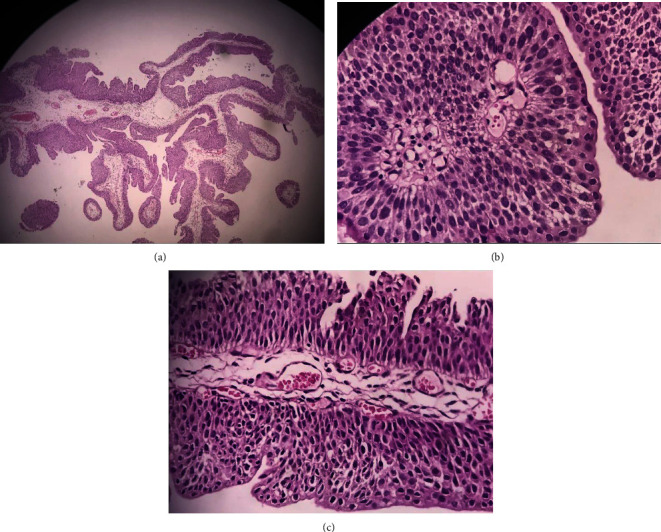
(a) Papillary urothelial neoplasm (H&E staining, low power field). (b, c) Low-grade noninvasive papillary urothelial carcinoma. The cytologic and architectural disorder is apparent with mild to moderate pleomorphism and scant mitotic figures (H&E staining, high power field).

Past medical history was unremarkable except for the urinary complaints mentioned. Her father was a heavy smoker who smoked in the house. He worked as a building painter and stocked chemical dyes at home. The only finding of physical examination was a mild suprapubic tenderness. Cystoscopy demonstrated a vegetative lesion on the right lateral aspect of the bladder. Transurethral resection of the bladder (TURB) was performed. Histological findings were “non-invasive papillary urothelial neoplasm composed of delicate and fused papillae lined by transitional cells with uniformly enlarged round to oval nuclei that show normal polarity and fine chromatin without visible nucleoli. The umbrella cells are present” (Figure 1). Thus, the pathology results confirmed the diagnosis of the papillary urothelial neoplasm of low malignant potential (PUNLMP). Tumor margins were clear with no detrusor involvement. Therefore, the chemotherapy regimen did not use as a part of treatment. The follow-up performed with cystoscopy every 6 months plus urine analysis and cytology every three months. No sign of recurrence was detected after one year.

## 3. Discussion

Based on WHO 2016 system, the UC classified into three subtypes: (1) papillary urothelial neoplasms of low malignant potential (PUNLMP), (2) noninvasive low-grade papillary urothelial carcinoma (NILGC), and (3) noninvasive high-grade papillary urothelial carcinoma (NIHGC) [3]. Any involvement of detrusor muscle signifies high-grade tumors [3]. It is worth mentioning that T1 tumors (invasion to subepithelial connective tissue, no muscle invasion) might be classified as high grade when they extend beyond lamina propria, invade lymph vasculature, or metastasize. Thus, non-muscle-invasive tumors should evaluate carefully after tumor resection [3]. Pediatric UC in 93.4% of patients is low grade. In terms of staging, 86.4% are TaN0M0, 7.0% are T1N0M0, and only 4.1% have higher stages [1].

Previous reports of UC could only detect a risk factor in 13.2-30% of cases. The most significant risk factor is tobacco exposure, which is reported in 7.8% of pediatric patients [1]. Other probable risk factors are environmental toxins (aromatic amines of hair dyes, industrial solvents, rubber), cancer medications (cyclophosphamide), congenital bladder abnormalities, and cancer-predisposing conditions (like Costello syndrome, Li-Fraumeni, Retinoblastoma, Turner syndrome) [1, 4]. Paner et al. declared lower Cyclin D2 hypermethylation and p53 overexpression among less than 20 years old compared to the adult population [4]. Oda et al. proposed that mutations in KRAS, and BRAF genes are possible predisposing factors [2]. However, previous studies suggested a latency time for a substance to exert its carcinogenic effects; our patient's exposure to second-handed smoke and industrial dyes might cause a synergistic effect. It is hypothesized that the higher incidence after the age of 13 shows probable endocrine-related pathogenesis of UC.

Isolated gross hematuria is the most common symptom present in about 75-80% of patients [1, 2]. Painless hematuria is more common, followed by irritative symptoms. However, abdominal/flank pain is less frequent [1]. About 90% of patients with UC have negative urine cytology [1]. Therefore, the negative results of cytology do not rule out the diagnosis of UC. Ultrasound is the primary imaging modality with 85-100% sensitivity, although computed tomography (CT) and MRI were also used in several studies [1]. Considering the pediatric population and risk of radiation, MRI is preferred over CT scans. Cystoscopy provides a definitive diagnosis and able the urologist for surgical resection.

TURB is the best option for the treatment with a low recurrence rate among pediatric patients. A partial bladder cystectomy is an option for those with high-grade tumors or when the cystoscope cannot access the tumor margins to achieve complete resection [1, 2]. Intravesical chemotherapy is advised for multifocal and/or high-grade tumors [1]. Neogi et al. reported a case of high-grade T2b-stage UC in a 4-year-old boy who recurred after four months of resection. He was treated with partial cystectomy and MVAC (methotrexate, vinblastine, Adriamycin, and cisplatin) chemotherapy and remained tumor-free for six months [5]. Patients with high-grade tumors and stages higher than TaN0M0 were more likely to recur [1].

There is no reliable guideline to define how to follow the patients and for how long. Based on Rezaee et al. study, most of the recurrences detected within the first year after TURB. They also found that the longest duration between the TURB and recurrence was 32 months. Therefore, they advised following low-grade cases less aggressively after three years of TURB. The recommended follow-up strategy is shown in Table 1 [1].

## 4. Conclusion

Painless hematuria in children and younger adults rarely suggests malignancy. Nevertheless, physicians need a high index of suspicion to diagnose bladder malignancies in the early stages. Early use of noninvasive ultrasound in persistent or intermittent hematuria seems reasonable. Physicians should also note that cytology has limited diagnostic value for UC. Regarding a safe follow-up approach, low-grade tumors are unlikely to recure after three years. Therefore, lower use of cystoscopy after three years of TURB is more likely to lower the adverse effects.

## Figures and Tables

**Table 1:** Follow-up strategy for low-grade pediatric UC.

	Urine analysis	Ultrasound	Cystoscopy
1st year after TURB every 3 months	+	+	+
2nd year after TURB every 6 months	+	+	+
3-5 years after TURB every 12 months	+	+	-/+
After 5 years	Patient education about red flags.		

## Data Availability

Data used to write this case report is accessible through contact with the corresponding author, Mottaghi M, Email: mmottaghi.3000@gmail.com.
